# Supplementary material for: Tensorpac: An open-source Python toolbox for tensor-based phase-amplitude coupling measurement in electrophysiological brain signals
Source: PLoS Comput Biol. 2020 Oct 29;16(10):e1008302. doi: 10.1371/journal.pcbi.1008302 (PMC7654762; doi:10.1371/journal.pcbi.1008302)
Supplement: Attachment — Submitted filename: Review of Combrisson et al. 2020 - PLoS Comp. Bio.pdf [file pcbi.1008302.s001.pdf]

Review of Combrisson et al. 2020

The manuscript by Combrisson and co-authors seeks to create an open-source toolbox for efficiently calculating phase amplitude coupling (PAC) in neural data sets. The authors identify two major limitations of the current PAC analysis pipeline: 1) the difficulties in interpreting PAC quantifications and 2) the computationally intense nature of PAC calculations on large data sets. To this effect, they implement a tensor-based Python package and parallelize computations for several known formulations of PAC quantification. The authors demonstrate that their package performs significantly faster than traditional vector / serial based approaches, and they provide an open-source toolbox that is available on GitHub.

In light of the general confusion surrounding PAC, its interpretation, and difficulties in its calculation, the manuscript and associated code by Combrisson et al. appear to be a valuable contribution to the field. In particular, a successfully implemented parallel approach would dramatically accelerate the generation of comodulograms, which necessarily require exhaustive pairwise testing of phase and amplitude frequencies.

I personally installed the Tensorpac package and conducted quick tests of many of the functionalities.

The central ‘Pac’ object as part of the pac.py class appears to be well constructed, and I tested several implementations of the code on different simulated data sets. It is generally very intuitive how to change the tuple inputs to create new Pac objects with different parameters, which is essential for the widespread adoption of this code package. The integrated surrogate generation for statistical comparisons is particularly useful.

The parallelization is accomplished using the joblib Python library, and I believe this to be a major contribution of the Tensorpac package. PAC calculations are by nature 'embarrassingly parallel’ and therefore the authors’ implementation of parallelized comodulogram generation is a timely and needed endeavor.

Taken together, I am enthusiastic about the publication of this work, and I believe it could be a useful contribution to the field of computational and systems neuroscience. However, there are some outstanding concerns to be addressed regarding the interpretation of measured PAC, validation on a real data set, and issues with reproducing some of the figures from the manuscript and Github repository.

Major

1. The primary factor that limits the overall utility of this toolbox stems from spurious PAC measurement due to the presence of non-sinusoidal oscillations in neural signals (eg. Lozano-Soldevilla et al., Front. Comp. Neuro, 2016; Gerber et al., PLoS One, 2016). The authors make use of either Hilbert transform or Morlet wavelet based approaches, but both are known to result in spurious PAC through creation of artifactual high frequency oscillations, harmonics in the spectral domain, and inaccuracies in modulating phase estimation. I appreciate that the authors cite some important literature on this topic (Kramer et al., 2008; Aru et al., 2015; and Cole et al., 2017), and they offer multiple PAC formulations which have varying susceptibility to artifactual measurements. However, I believe a more nuanced discussion of this is warranted in the manuscript itself, especially since widespread adoption of this code will necessarily invoke these arguments.

As this has been a contentious subject in the field for some time now, there are several review articles that provide guidance on how to interpret spurious versus genuine PAC (Jensen et al., eNeuro, 2017; Cole and Voytek, Trends in Cog. Sci., 2017). While opinions vary, a common approach is to verify genuine oscillatory

peaks in the power spectral densities (PSD) before calculating PAC. For example, a discussion of the author's implementation of PSD calculation and the triangular sweep (Fig. 7) to possibly assess true oscillatory peaks could be useful in this regard.

Additionally, several alternative approaches for measurement of these waveform phenomena have been proposed to try to combat spurious PAC measurements. These include bispectral approaches (Kovach et al., NeuroImage, 2018), autoregressive models (La Tour, PLoS Comp. Bio., 2017), and purely time domain measurements (Cole and Voytek, J. Neurophys, 2019). It could also be helpful to highlight the contributions of the authors' work in light of these alternative approaches.

I realize that the authors are not responsible for the deficiencies in the measurement of PAC itself, and their primary goal was to make a toolbox for efficient quantification of PAC, but a discussion of the above topics would significantly improve the manuscript and provide readers with a context for how to interpret the output results of the Tensorpac toolbox.

2. I also believe that the general utility of the Tensorpac library would be best demonstrated on a real data set. I appreciate that the authors built several useful features for simulating PAC containing signals, but as widespread adoption of the code will be predicated on application to real neural data, it would be very helpful to see known PAC results replicated with this toolbox. This should be relatively straightforward for the authors to do, given their previous experience with PAC in human iEEG data (Combrisson et al., NeuroImage, 2017).

Minor

1. I received different results than those in the main manuscript when I ran the listed code for Fig 3 (tensorpac / paper / fig_3_compare_pac.py). For reference, I checked this in both Jupyter Notebooks and VSCode, which are both commonly used Python compilers. In particular, the scale of 'ndPAC' seems to be completely different than in the main manuscript:

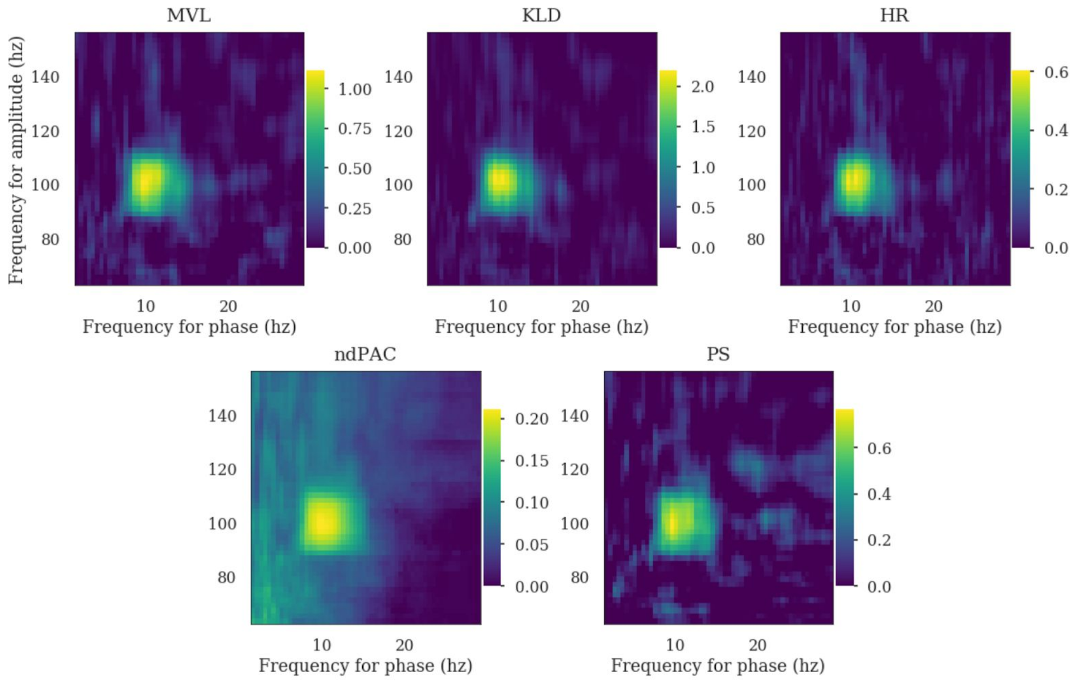

2. Related to the above point, I copied and ran the "Code snippet & illustration" (from README.rst) and received different results than those shown on the front page of the GitHub repository. I have attached the output from Jupyter Notebooks with associated code below in case that is helpful for resolving this issue:

```
from tensorpac import Pac
from tensorpac.signals import pac_signals_tort

# Dataset of signals artificially coupled between 10hz and 100hz :
n_epochs = 20
n_times = 4000
sf = 512.  # sampling frequency

# Create artificially coupled signals using Tort method :
data, time = pac_signals_tort(f_pha=10, f_amp=100, noise=2, n_epochs=n_epochs,
                              dpha=10, damp=10, sf=sf, n_times=n_times)

# Define a PAC object :
p = Pac(idpac=(6, 3, 0), f_pha=(2, 20, 1, 1), f_amp=(60, 150, 5, 5))
# Filter the data and extract PAC :
xpac = p.filterfit(sf, data, n_perm=20)

# Plot your Phase-Amplitude Coupling :
p.comodulogram(xpac.mean(-1), title='Contour plot with 5 regions',
               cmap='Spectral_r', plotas='contour', ncontours=5)

p.show()
```

```
Phase Amplitude Coupling object defined
    Extract phases (n_pha=17) and amplitudes (n_amps=17)
    true PAC estimation using Gaussian Copula PAC (Ince et al. 2017)
    compute surrogates (Time lag (Canolty et al. 2006), 20 permutations)
    Infer p-values at p=0.05
```

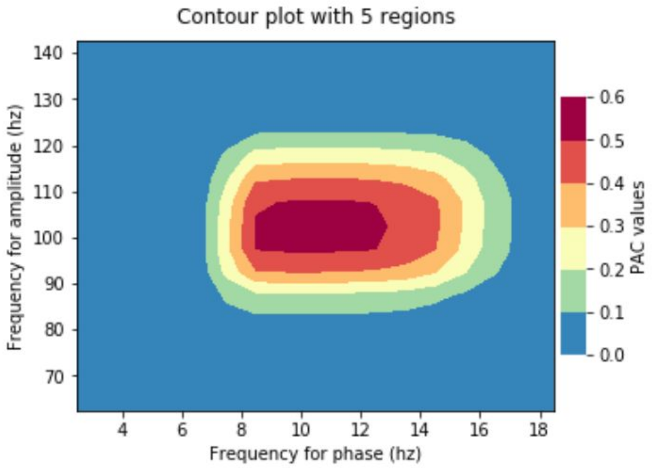

3. Figure 8B title should read vector / tensor (instead of tensor / vector) unless I am mistaken.

4. Gaussian copula PAC is included as a method in the Tensorpac package, and is used in the example on the front page of the GitHub repository, but its formulation is not listed in the manuscript and does not appear in Fig3.

5. While this is not a pressing issue, the authors could in many cases consider using Numba/Cython to speed up some processing aspects of the Python code. Could consider this in future updates to the library.

Proofreading

1. Pg. 5 -- “Extracting the phase and the amplitude using the absolute value and the phase of the Hilbert transform applied on these filtered signals.” - revise this sentence

2. Pg. 6 -- “Height-Ration” - typo

3. Pg. 11 “taken a an” - typo
