# Supplementary material for: Tensorpac: An open-source Python toolbox for tensor-based phase-amplitude coupling measurement in electrophysiological brain signals
Source: PLoS Comput Biol. 2020 Oct 29;16(10):e1008302. doi: 10.1371/journal.pcbi.1008302 (PMC7654762; doi:10.1371/journal.pcbi.1008302)
Supplement: Attachment — Submitted filename: response_to_reviewers.pdf [file pcbi.1008302.s002.pdf]

Marseille, August 4th, 2020

Revision note

Manuscript Ref: PCOMPBIOL-D-20-00589

Dear Editors,

We are pleased to submit a revised version of our manuscript entitled "*Tensorpac : an open-source Python toolbox for tensor-based Phase-Amplitude Coupling measurement in electrophysiological brain signals*" by Etienne Combrisson, Timothy Nest, Andrea Brovelli, Robin A.A. Ince, Juan LP Soto, Aymeric Guillot and Karim Jerbi, currently under consideration for publication in PLoS Comp Biol as a software paper.

All the comments and questions of the reviewers have been addressed, point-by-point, in our response letter, and all suggested improvements to the API and online tutorial have been implemented. We feel that all of the changes and new extensions to the package have substantially improved the quality and clarity of our manuscript and utility of the software.

We hope that this re-submission will be positively evaluated and we remain at your disposal should you require any further information.

Best regards,

Etienne Combrisson & Karim Jerbi

Point-by-point response to reviews

Ms. No.: PCOMPBIOL-D-20-00589

Title: *Tensorpac : an open-source Python toolbox for tensor-based Phase-Amplitude Coupling measurement in electrophysiological brain signals*

Authors: Etienne Combrisson, Timothy Nest, Andrea Brovelli, Robin A.A. Ince, Juan LP Soto, Aymeric Guillot and Karim Jerbi

In the following, we provide point-by-point responses to all the comments and questions raised by Reviewers #1, #2 and #3, respectively. For the sake of clarity, the reviewers' comments are in plain text, our responses are in bold font, and additions/changes made to the manuscript are presented here in blue.

REVIEWER 1

Summary

> *This paper describes a Python based toolbox to estimate, most of all, phase-amplitude coupling. This area of research has recently attracted much interest and therefore the toolbox is potentially useful for a large number of researchers. The paper is very well written and, perhaps with one exception to be discussed below, free of errors. In addition to implementing existing methods, the authors developed a new visualization technique shown in Fig.6B which is really nice. I still have some objections which should be addressed by the authors.*

We thank the reviewer for this positive feedback about both the paper and the toolbox. In the following we address all the issues raised by this reviewer. We also provide in this link[1] all the scripts that can be used to reproduce the figures included in the revised manuscript including the ones included in our answers to the reviewer.

Major

> *1. The authors present a formula to estimate a threshold written as 2 × ((1 −)^−1)^2. For a z-scored variable, the threshold would be the square root of that. Perhaps it is meant to be the threshold for the square of the z-scored MVL value, but here the authors should be more specific of what exactly is thresholded. Also, writing the exponent -1 in the formula after the brackets for the argument of erf is confusing. That looks like 1/erf(1-p). Finally, to my opinion this z-scoring and the statistical interpretation as a Gaussian distribution with zero mean and unit standard deviation doesn't make sense. The distribution of MLV under the null hypothesis isn't even remotely close to a Gaussian distribution. I don't blame the authors. They just implemented methods proposed by others, but a warning remark would be helpful.*

We thank the reviewer for this useful comment about the statistical threshold proposed by Ozkurt, 2012. In their paper, the authors made the assumption that the amplitude is normally distributed (zero mean and unit variance), probably because the probability density function (pdf) of a Gaussian variable is well defined and then, much easier to work with. Therefore, they proposed a z-score normalization of the amplitude to get closer to the assumptions. Importantly, this normalization is performed before computing the PAC. In addition, they also assumed that the phase is

[1] https://github.com/EtienneCmb/tensorpac/tree/master/paper

uniformly distributed between -pi and pi. In order to check how those assumptions hold, we simulated data with a coupling between a 10hz phase and 100hz amplitude and then binned the extracted phase and amplitude. The assumption about the phase seems to be reasonable (see figure below, blue distribution). The non-normalized amplitude is plotted in red and the orange distribution represents the binned amplitude after the z-score normalization. While the orange distribution seems to be mostly Gaussian, we acknowledge that, even with simulated data, a Poisson distribution would probably better fit with how amplitudes are actually distributed.

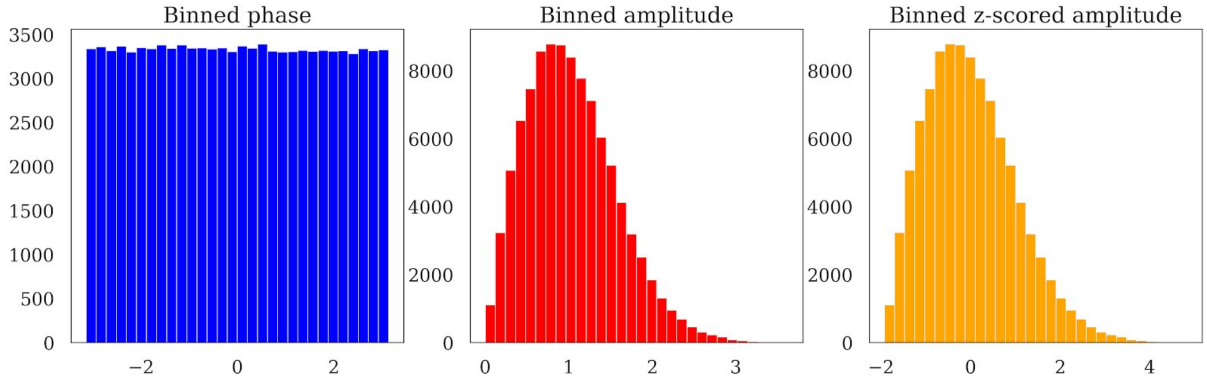

But as the reviewer noted, this is how the method was originally proposed. However, the description of the ndPAC in the original manuscript was indeed not very clear. We improved the text in our manuscript by specifying the assumptions of the method to provide a better explanation of how the threshold works :

"In order to find this threshold, the amplitude is assumed to be normally distributed (zero mean and unit variance) and the phase is assumed to be uniformly distributed between -π and π. To this end, the mean and deviation across time points of the amplitude are used to perform a z-score normalization in order to approximate the original assumptions. Finally, every value of coupling exceeding this threshold is considered as reliable, at a given confidence level. Otherwise, if the value of coupling is below this threshold it is set to zero."

About the equation, the reviewer was right, the -1 exponent was misplaced. We moved it in the equation to reflect the fact that it is the inverse error function (erf^-1(1 - p)) and not the inverse <u>of</u> the error function erf(1-p)^-1. In addition, the number of time points *N* was also missing compared to the original publication and has been corrected in the revised manuscript.

> 2. The corrected PAC shown in Fig.4C is constructed by subtracting the mean and dividing by the standard deviation of the surrogates, right? At least, this is how understand the sentence "... usually by subtracting the mean and divide by the deviation of this distribution" on p.13. But why are the results so small? [...]

PAC computed with the MVL and normalized by the surrogates have values that are indeed small compared to those obtained with the ndPAC in Fig3. The z-score normalization is frequently used and is the normalization type proposed for the MVL by Canolty 2006. However, this normalization is performed in order to correct the PAC using the distribution of surrogates. In other words, this specific normalization is made for removing PAC that could be obtained by chance. Therefore, the normalization is performed using the mean and deviation of PAC values obtained by chance. On the other hand, as explained above for the ndPAC, the z-score normalization is performed in order to approximate the assumptions of Gaussianity. And this z-score is performed only on the amplitude (subtracting the mean amplitude across time points and dividing by the deviation also obtained across time points). Finally, the PAC is computed using this z-score version of the amplitude. This explains why PAC computed with the MVL and normalized by the surrogates have much smaller values (Fig4) than those obtained with the ndPAC in Fig3.

Finally, the illustration of the corrected PAC using the distribution of surrogates (Fig4) was not performed using a z-score but instead, a subtraction and division by the mean of the surrogates (as explained in the legend).

[...] Not any of the shown results would survive the threshold discussed above. As stated above, I doubt that the threshold is based on accurate statistics, but I would still expect values in the order of 1 even when the null hypothesis is true, and I would expect very high values for real effects like in Fig.3 ndPAC. If it is not normalized here, why does the difference between Fig.4.A and Fig.4.B has values larger than 0.3 in the central peak?

As the reviewer noted, ndPAC values should be comprised between [0, 1] which was not the case in the original manuscript. This issue has been fixed since the initial submission of our article and the Fig3 has been updated accordingly. In fact, this update was linked to an issue we addressed that was raised by a Tensorpac user who also proposed a patch (see #PR9[2]).

To provide an insight of how this threshold operates, we simulated a coupling between a 10hz phase and 100hz amplitude (illustrative single trial). We slightly modified the ndPAC function so that it returns the non-thresholded PAC, the threshold at $p<0.05$ and the resulting threholded PAC. As the reviewer can see below, the threshold is removing parts of the comodulogram where the probability of having a coupling is very low (e.g high frequency phase).

[2] https://github.com/EtienneCmb/tensorpac/pull/9

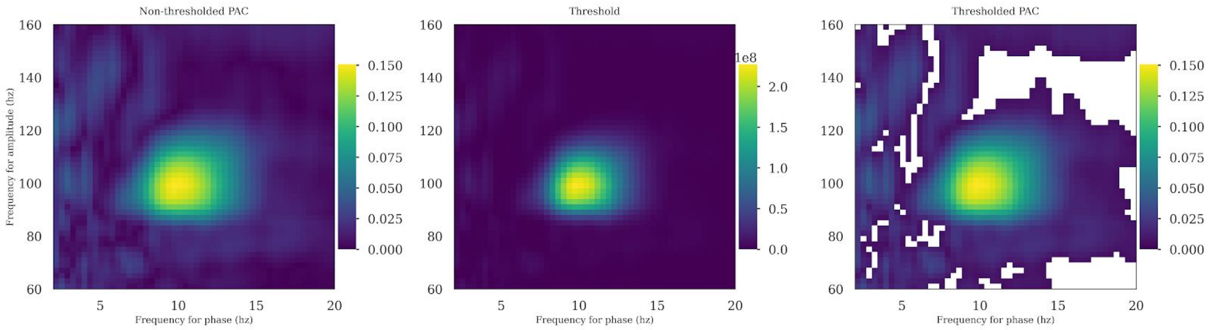

Overall, we share the doubts of the reviewer about the accuracy of this method that uses a statistical threshold. In addition, this insight is performed in a controllable context of simulated data, it is probably even worse in a real data scenario where the inter-trial coherency is probably lower. Although the ndPAC approach has not been largely adopted by the community, we believe that having this method implemented in our package is good for comparison with other methods.

> *Finally, I also wonder whether MVL is interpretable without any normalization because the results will depend on overall signal amplitude.*

We thank the reviewer for this comment because indeed, using the MVL without the normalization might be problematic, for example when it comes to comparing conditions, since the MVL is amplitude dependent. To make it clear for the user, we added a warning in the code when the MVL is chosen without a normalization (see #a29f0dd[3])

Minor

> *> 1. As far as I know, what is called PLV in this paper, is usually called ITC (inter trial coherence) and what is called PS here I would call PLV. Perhaps terminology varies. Just check.*

We thank the reviewer for this comment. To untangle what we think are the differences between those measures :

- **ITC (which is also called the Phase-Locking Factor) is a measure of phase consistency over trials**
- **PLS (Phase Locking Statistics) seems to be a family of statistical measures of phase consistency which comprises :**

[3] https://github.com/EtienneCmb/tensorpac/commit/a29f0dd69ca93bfc485be784b6897cec6d46b828

- **PLV measures the phase consistency over trials but between a pair of electrodes (Lachaux, 1999).**
- **S-PLS (single-trial PLS) also measures the consistency of phase between a pair of electrodes but this time it is computed across time, over small sliding windows (Lachaux, 1999)**

One subtlety in the case of measuring PAC is that it is the PLV between the low frequency phase and the phase of the high frequency amplitude for a single recording site. We agree with the reviewer that this is slightly different from the original definition of the PLV. Therefore, we have changed in the toolbox, in the figures and in the text the name of PLV for Inter-Trial Coherence (ITC) and the Phase-Synchrony is now the PLV.

> *2. Last equation of section "Kullback-Leibler distance:" Brackets are missing. It should read log(P(k)) and not log(PK).*

This has been corrected in the revised version.

3. p.11 "This distribution is then used to compute PAC using either the KLD." Or the normalized MVL?

Here, the reviewer is referring to the distribution of amplitudes binned according to phase values. This binning operation is really inherent to the KLD and HR approaches and is actually what links the phase and the amplitude together (Tort, 2010). The sentence was not complete and has been updated with :

“This distribution is then used to compute PAC using either the modulation index either the heights-ratio“

We sincerely thank this reviewer for all the helpful and detailed comments on the manuscript and the software.

REVIEWER 2

Summary

The manuscript by Combrisson and co-authors seeks to create an open-source toolbox for efficiently calculating phase amplitude coupling (PAC) in neural data sets. The authors identify two major limitations of the current PAC analysis pipeline: 1) the difficulties in interpreting PAC quantifications and 2) the computationally intense nature of PAC calculations on large data sets. To this effect, they implement a tensor-based Python package and parallelize computations for several known formulations of PAC quantification. The authors demonstrate that their package performs significantly faster than traditional vector / serial based approaches, and they provide an open-source toolbox that is available on GitHub.

In light of the general confusion surrounding PAC, its interpretation, and difficulties in its calculation, the manuscript and associated code by Combrisson et al. appear to be a valuable contribution to the field. In particular, a successfully implemented parallel approach would dramatically accelerate the generation of comodulograms, which necessarily require exhaustive pairwise testing of phase and amplitude frequencies.

I personally installed the Tensorpac package and conducted quick tests of many of the functionalities.

The central 'Pac' object as part of the pac.py class appears to be well constructed, and I tested several implementations of the code on different simulated data sets. It is generally very intuitive how to change the tuple inputs to create new Pac objects with different parameters, which is essential for the widespread adoption of this code package. The integrated surrogate generation for statistical comparisons is particularly useful.

The parallelization is accomplished using the joblib Python library, and I believe this to be a major contribution of the Tensorpac package. PAC calculations are by nature 'embarrassingly parallel' and therefore the authors' implementation of parallelized comodulogram generation is a timely and needed endeavor.

Taken together, I am enthusiastic about the publication of this work, and I believe it could be a useful contribution to the field of computational and systems neuroscience. However, there are some outstanding concerns to be addressed regarding the interpretation of measured PAC, validation on a real

data set, and issues with reproducing some of the figures from the manuscript and Github repository.

We thank the reviewer for this very positive feedback as well as for taking the time to install and test the functionalities of our software. In the following we address all the issues raised by this reviewer. Note that we also provide in this link[4] all the scripts that can be used to reproduce the figures included in the revised manuscript including the ones included in our answers to the reviewer.

Major

> 1) The primary factor that limits the overall utility of this toolbox stems from spurious PAC measurement due to the presence of non-sinusoidal oscillations in neural signals (eg. Lozano-Soldevilla et al., Front. Comp. Neuro, 2016; Gerber et al., PLoS One, 2016). The authors make use of either Hilbert transform or Morlet wavelet based approaches, but both are known to result in spurious PAC through creation of artifactual high frequency oscillations, harmonics in the spectral domain, and inaccuracies in modulating phase estimation. I appreciate that the authors cite some important literature on this topic (Kramer et al., 2008; Aru et al., 2015; and Cole et al., 2017), and they offer multiple PAC formulations which have varying susceptibility to artifactual measurements. However, I believe a more nuanced discussion of this is warranted in the manuscript itself, especially since widespread adoption of this code will necessarily invoke these arguments.

As this has been a contentious subject in the field for some time now, there are several review articles that provide guidance on how to interpret spurious versus genuine PAC (Jensen et al., eNeuro, 2017; Cole and Voytek, Trends in Cog. Sci., 2017). While opinions vary, a common approach is to verify genuine oscillatory peaks in the power spectral densities (PSD) before calculating PAC. For example, a discussion of the author's implementation of PSD calculation and the triangular sweep (Fig. 7) to possibly assess true oscillatory peaks could be useful in this regard.

Additionally, several alternative approaches for measurement of these waveform phenomena have been proposed to try to combat spurious PAC measurements. These include bispectral approaches (Kovach et al., NeuroImage, 2018), autoregressive models (La Tour, PLoS Comp. Bio., 2017), and purely time domain measurements (Cole and Voytek, J. Neurophys, 2019). It could also be helpful to highlight the contributions of the authors' work in light of these alternative approaches.

---

[4] https://github.com/EtienneCmb/tensorpac/tree/master/paper

I realize that the authors are not responsible for the deficiencies in the measurement of PAC itself, and their primary goal was to make a toolbox for efficient quantification of PAC, but a discussion of the above topics would significantly improve the manuscript and provide readers with a context for how to interpret the output results of the Tensorpac toolbox.

We thank the reviewer for this very useful comment for pointing us to the very important literature on the risks of (and ways to deal with) spurious PAC. Overall, we agree with the comment and we also believe that, when introducing our toolbox, it is important to highlight these conceptual limitations even though, as the reviewer mentions, we are not responsible for the deficiencies inherent to PAC measurement. We went through the recommended papers and decided to incorporate a new paragraph in the revised manuscript with a more extensive discussion of spurious PAC:

" **4. The case of spurious cross-frequency coupling**

Previous research has established that non-oscillations, non-stationary signals and sharp waveforms (e.g the mu rhythm in sensorimotor areas) can all produce spurious PAC even in the absence of true phase-amplitude coupling (Lozano-Soldevilla et al. 2016; Gerber et al., 2016; Aru et al. 2015; Kramer et al., 2008). While considering non-oscillations is an important aspect of brain data analysis (Jensen et al., 2017; Cole and Voytek, 2017), the fact that it can also produce non-existent couplings is problematic for future work on cross-frequency coupling. Most of the standard PAC methods rely on an instantaneous estimation of the phase and amplitude using Hilbert transform or Morlet wavelets. However, both approaches can introduce spurious PAC from sharp waveform features. A few methods, not based on instantaneous estimations of phase and amplitude, have been introduced to tackle the issue of spurious PAC. These include for example auto-regressive models (Dupre La Tour et al., 2017) and bispectral approaches (Kovach et al. 2018). The default instantaneous signal decompositions in phase and amplitude included in Tensorpac at the time of publication do not solve the ongoing debate on spurious versus genuine PAC, nevertheless the package provides a robust and convenient framework for the incorporation of additional PAC methods which may be developed to address this issue in the future.

More generally, when using Hilbert or wavelets, several guidelines and precautions have been proposed in order to limit spurious PAC and control the accuracy of an estimation (Aru et al. 2015). First, a visual inspection of the time-series could be performed in order to exclude non-stationary signals. In addition, a clear peak at the phase frequency should be visible in the PSD to warrant further exploration of PAC (this can be examined in Tensorpac using *tensorpac.utils.PSD*). Still, the Fourier transform decomposes a signal in a sum of sines and cosines which means that it can only partially capture non-oscillatory features (Lozano-Soldevilla et al. 2016; Cole and Voytek, 2017). Instead, one might consider using a matching pursuit algorithm or an empirical mode decomposition since both do not make the assumption of a sinusoidal basis (Cole and Voytek, 2017). In addition to these two

alternatives, a method based on cycle-by-cycle analysis has recently been proposed in order to extract temporal features (Cole and Voytek, 2019). In principle, if a peak in the PSD is observed at the frequency of the phase, the width of this peak should be used to then extract the instantaneous phase. When filtering the amplitude, the width of the frequency band should be large enough to contain the sidebands of the lower frequency band. In practice it means that if the band used for low frequency phase is [8, 10]Hz, the bandwidth of the amplitude should be at least 20Hz. The presence of coupling can also be further explored by binning the amplitude according to phase slices (*tensorpac.utils.BinAmplitude*). In absence of coupling the distribution should be uniform while in presence of coupling, this distribution of amplitude should be closer to a normal distribution. Tensorpac also includes the possibility to realign time-frequency representations based on a phase peak (*tensorpac.utils.PeakLockedTF*). If a coupling exists, a rhythmic pattern of higher frequency (e.g. gamma) amplitude bursts should be observed."

> *2) I also believe that the general utility of the Tensorpac library would be best demonstrated on a real data set. I appreciate that the authors built several useful features for simulating PAC containing signals, but as widespread adoption of the code will be predicated on application to real neural data, it would be very helpful to see known PAC results replicated with this toolbox. This should be relatively straightforward for the authors to do, given their previous experience with PAC in human iEEG data (Combrisson et al., NeuroImage, 2017).*

We thank the reviewer for this comment. We have now created a "Tutorials" section in the gallery of examples. This section now includes a full introduction to PAC analysis[5] using sample intracranial EEG (iEEG) data from our previous publication (Combrisson et al. 2017), as suggested by the reviewer. This tutorial includes computing the Inter-Trial Coherence, Power Spectrum Density, Event-Related PAC, alignment of time-frequency representation based on phase peak, computing PAC with statistics (corrected for multiple comparisons) such as the estimation of the preferred-phase. We also added a sentence to section *5. Documentation and API provision* **:**

"It also features a gallery of examples, built with sphinx-gallery, which demonstrate the use of Tensorpac's main classes and functions and a step-by-step tutorial based on real intracranial EEG data recorded during a center-out motor task [19]"

---

5 https://etiennecmb.github.io/tensorpac/auto_examples/tuto/plot_real_data.html#sphx-glr-auto-examples-tuto-plot-real-data-py

Minor

1) I received different results than those in the main manuscript when I ran the listed code for Fig 3 (tensorpac / paper / fig_3_compare_pac.py). For reference, I checked this in both Jupyter Notebooks and VSCode, which are both commonly used Python compilers. In particular, the scale of 'ndPAC' seems to be completely different than in the main manuscript:

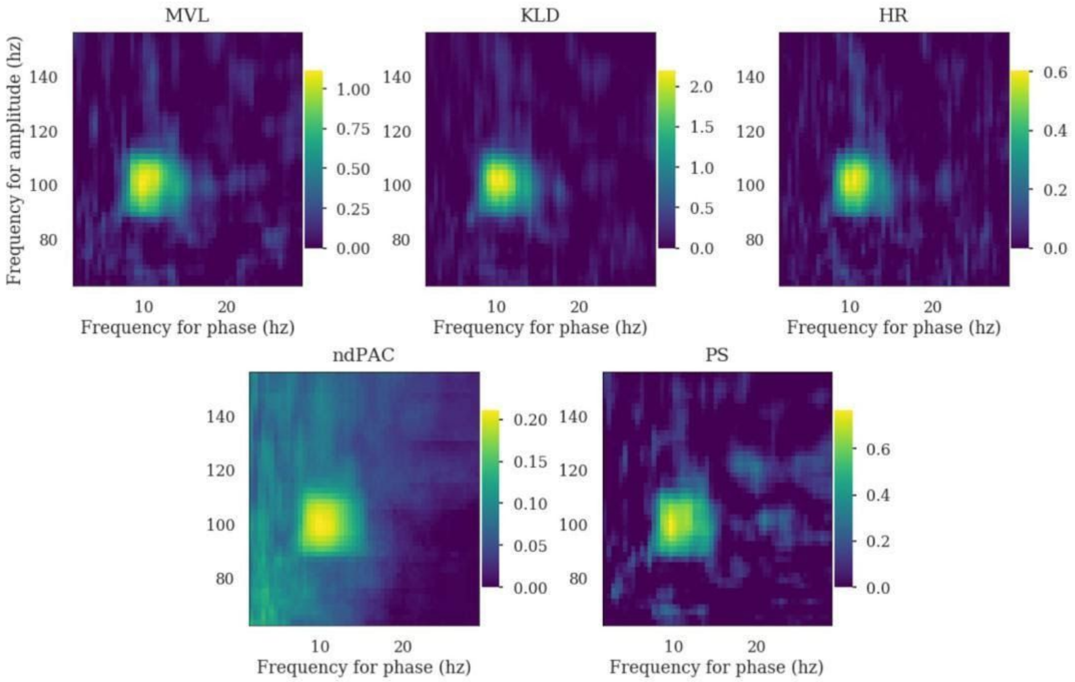

We thank the reviewer for attempting to reproduce this figure. The reviewer is right in noting that the code for figure 3 does not produce the figure in the original manuscript. This is because the ndPAC scaling has been corrected in Tensorpac since the initial submission: a Tensorpac user found an issue with the implementation of the ndPAC (see #PR9[6]). Outputs were not correctly normalized which was producing very high values. This behavior has been fixed since then and the figure in the manuscript has also been updated.

In addition to this point, in Reviewer 1 pointed out to us that the phase synchrony measure is most known in the litterature as the "Phase Locking Value" (PLV) (Penny et al. 2008, Lachaux et al. 1999). Therefore, in the toolbox, in the manuscript and in the title of the figure phase synchrony (PS) has been renamed PLV and the full names of each method have been updated.

Finally, we also included the Gaussian-Copula PAC to the comparisons in this figure. The revised manuscript now contains the following updated figure :

[6] https://github.com/EtienneCmb/tensorpac/pull/9/

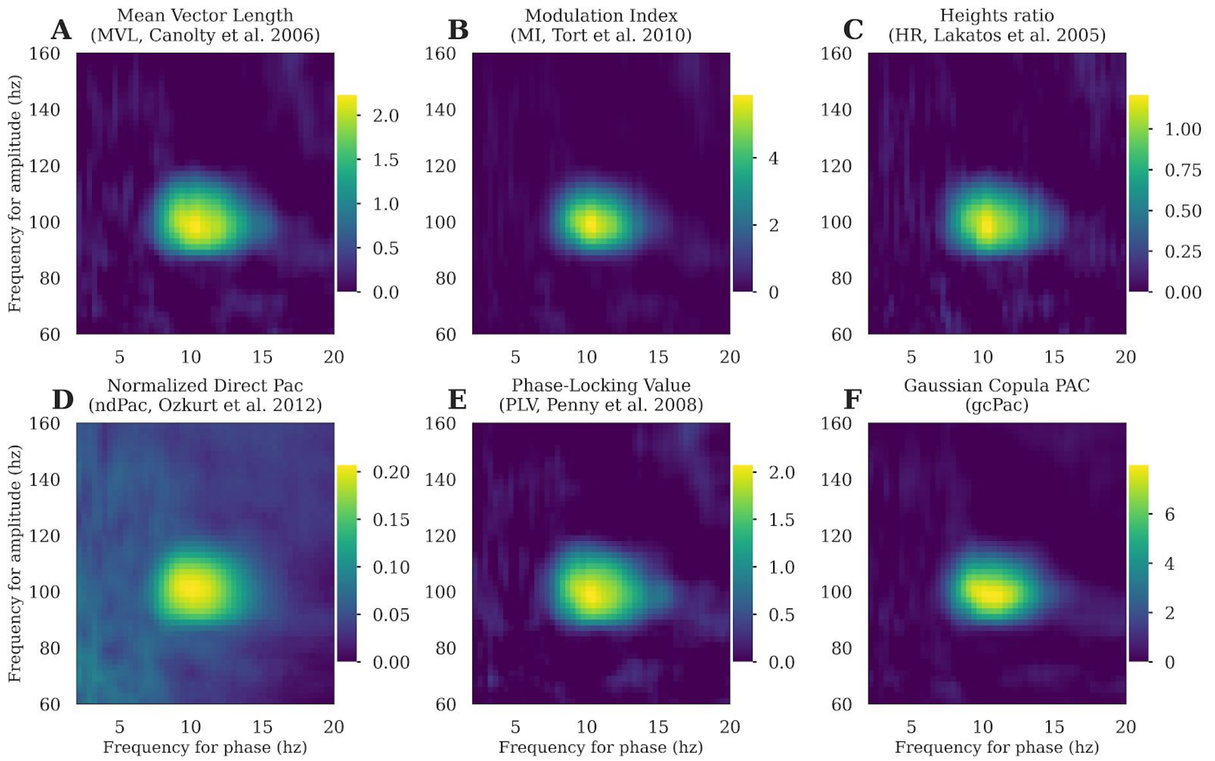

> 2) Related to the above point, I copied and ran the “Code snippet & illustration” (from README.rst) and received different results than those shown on the front page of the GitHub repository. I have attached the output from Jupyter Notebooks with associated code below in case that is helpful for resolving this issue:

```
from tensorpac import Pac
from tensorpac.signals import pac_signals_tort

# Dataset of signals artificially coupled between 10hz and 100hz :
n_epochs = 20
n_times = 4000
sf = 512.  # sampling frequency

# Create artificially coupled signals using Tort method :
data, time = pac_signals_tort(f_pha=10, f_amp=100, noise=2, n_epochs=n_epochs,
                              dpha=10, damp=10, sf=sf, n_times=n_times)

# Define a PAC object :
p = Pac(idpac=(6, 3, 0), f_pha=(2, 20, 1, 1), f_amp=(60, 150, 5, 5))
# Filter the data and extract PAC :
xpac = p.filterfit(sf, data, n_perm=20)

# Plot your Phase-Amplitude Coupling :
p.comodulogram(xpac.mean(-1), title='Contour plot with 5 regions',
               cmap='Spectral_r', plotas='contour', ncontours=5)

p.show()
```

```
Phase Amplitude Coupling object defined
    Extract phases (n_pha=17) and amplitudes (n_amps=17)
    true PAC estimation using Gaussian Copula PAC (Ince et al. 2017)
    compute surrogates (Time lag (Canolty et al. 2006), 20 permutations)
    Infer p-values at p=0.05
```

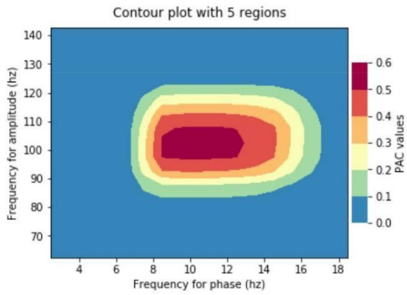

Indeed, this is a good point. We updated the figure and the code included in the README, as well as the example in the gallery[7]. To the best of our knowledge, there is no existing solution to keep an up-to-date README that keeps track of the flow of commits. Maybe a lasting solution we could consider in the future is to construct the README.rst on CircleCI and then push it back to the Github repository.

> *3) Figure 8B title should read vector / tensor (instead of tensor / vector) unless I am mistaken.*

Indeed, it is of course the tensor implementation that takes less computing time to run. The title of Fig8B has been updated accordingly.

> *4) Gaussian copula PAC is included as a method in the Tensorpac package, and is used in the example on the front page of the GitHub repository, but its formulation is not listed in the manuscript and does not appear in Fig3.*

Indeed, Tensorpac also includes PAC and ERPAC methods based on mutual-information which were not included in the manuscript. The original plan was to continue working on all of the possibilities that the versatility of the mutual-information can bring to the estimation of PAC and separate this methodological work from the Tensorpac toolbox. However, since reviewers 2 and 3 both highlighted it, we decided to include the Gaussian-Copula PAC (gcPAC) as well as the Gaussian-Copula ERPAC (gcERPAC) methods to the manuscript. Figures 3 and 5 now include respectively the gcPAC and gcERPAC.

We added the following paragraph to section 1.3, describing the gcPAC computed across time-points :

"**Gaussian-Copula PAC :** Mutual Information (MI) can be used to quantify pairwise statistical dependence between many different types of variables on a common and meaningful effect size scale (bits) (Cover & Thomas, 1991). However, MI can be difficult to estimate in practice as it requires sampling the full joint distribution of the two considered variables, each of which can themselves be multivariate. Gaussian-Copula Mutual Information (GCMI) is a recently proposed semi-parametric estimation technique (Ince et al. 2017) which has some advantages for estimating MI from neural data. GCMI exploits the fact that mutual information is copula entropy: MI does not depend on the marginal distributions of the variables, but only on the copula function which describes their dependence. GCMI therefore first transforms the inputs to be standard normal. This copula-normalisation step involves calculating the inverse standard normal cumulative density function (CDF) value of the

---

7 https://etiennecmb.github.io/tensorpac/auto_examples/pac/plot_readme.html#sphx-glr-auto-examples-pac-plot-readme-py

empirical CDF value of each sample, separately for each input dimension, before using a parametric, bias-corrected, Gaussian MI estimator. This provides a lower bound estimate of the MI, without making any assumption on the marginal distributions of the input variables. GCMI is rank-based, robust and scales well with multidimensional variables as the Gaussian model reduces the curse of dimensionality that faces other methods such as those involving binning.

In the case of PAC, the phase $\Phi(t)$ and the amplitude $a(t)$ are still extracted using the Hilbert transform or using wavelets. The rank normalization is first applied on the amplitude $a(t)$. To preserve the cyclic nature of phase, it is represented as points on the unit circle of the complex plane, represented as a 2d variable for GCMI. This 2d variable is built by concatenating the sine and cosine of the phase $\Phi(t)$, $([sin(\Phi(t)), cos(\Phi(t))])$ projecting it into the unit circle (or alternatively by normalising away the amplitude of the complex value). The copula normalization procedure is then applied to each dimension of this 2d variable. PAC is measured as the GCMI between the copula-normalized 1d high-frequency amplitude and the 2d representation of low-frequency phase :

$$gcPAC = I(a(t);\ [sin(\Phi(t)),\ cos(\Phi(t))])$$

Since the gcPAC is rank-based, it is, by construction, invariant to overall amplitude shifts which means that a power increase does not lead to an increase of coupling. Note that measuring PAC using mutual-information has already been proposed using nearest-neighbour estimators (Martínez-Cancino et al., 2019). Similarly to the other measures, Tensorpac provides a tensor-based implementation of the GCMI computed between two continuous variables."

In addition, we added to section 1.5, the description of the gcERPAC, this time computed across trials :

"Similarly to the gcPAC, we also introduce the Gaussian-Copula Event-Related PAC which this time measures the information shared by the phase and the amplitude across trials, at each time point."

> *5) While this is not a pressing issue, the authors could in many cases consider using Numba/Cython to speed up some processing aspects of the Python code. Could consider this in future updates to the library.*

This is a suggestion that we were actually considering, in particular for the PAC computation functions. Therefore, we followed the reviewer's advice and have now added to Tensorpac a Numba implementation for some of the main PAC methods (MVL, MI, HR, ndPAC, PLV). Please, find below a figure illustrating the computing time comparison between the tensor and the Numba implementations. As the reviewer can see, some methods could actually be faster with Numba (MVL, ndPAC and PLV), while the MI and HR are two times slower with Numba. That said, we have to acknowledge

that for these two methods, a better Numba compliant implementation may be possible. Using Numba with the Gaussian-Copula PAC was not possible since it uses some specific functions of scipy that don't seem to be supported for the moment. This can be added in the future.

However, we are facing one main issue to have a deeper integration of Numba inside Tensorpac. Numba doesn't seem to marry well with the parallel computing of Joblib. In particular, it seems that when performing distributing the code, the input arrays change flags from a "writable" to "read only" mode and then Numba complains that there is no matching definition for read only compiled function. We would appreciate it if the reviewer has an experience about this point to fix the combination between Numba and parallel computing.

Overall, we agree with the reviewer that there is space for more Numba/Cython inside Tensorpac, beyond the PAC functions we added. One example is for generating the permutations. But we should also consider that it introduces some potential bugs, troubles and limitations that we should consider in a long-term developpement.

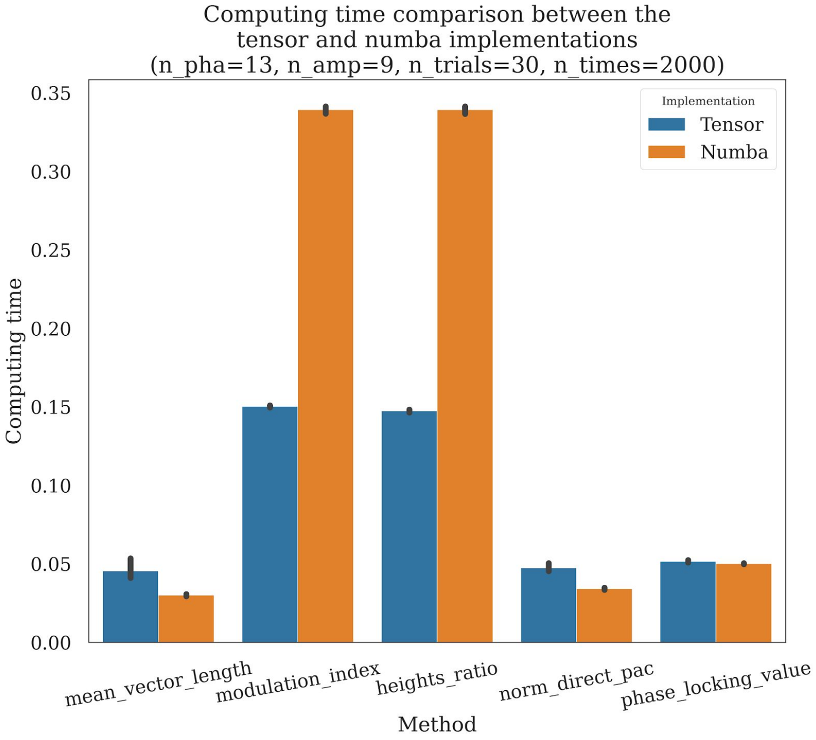

Proofreading

> *1) Pg. 5 -- "Extracting the phase and the amplitude using the absolute value and the phase of the Hilbert transform applied on these filtered signals." - revise this sentence*

We rephrased this sentence to :

"The phase and the amplitude are respectively obtained by taking the angle and the absolute value of the Hilbert transform applied to the complex analytic filtered signals."

> *2) Pg. 6 -- "Height-Ration" - typo*

Corrected.

> *3) Pg. 11 "taken a an" - typo*

Corrected.

We sincerely thank this reviewer for all the helpful and detailed comments on the manuscript and the software.

REVIEWER 3

Summary

> *> The authors present an open-source toolbox 'tensorpac' for calculation of phase-amplitude coupling (PAC) measures. The toolbox features large speed gains in PAC-computation due to the usage of tensors in contrast to vector-based computation. Given clarifications regarding advantages with respect other existing toolboxes for PAC and statistical analysis, as well as extending the documentation, I believe this a valuable methodological contribution.*

We thank the reviewer for the overall positive comment. In the following we address all the issues raised by this reviewer. We also provide in this link[8] all the scripts that can be used to reproduce the figures included in the revised manuscript including the ones included in our answers to the reviewer.

Major

The authors list 4 points as main advantages of their toolbox:

> *> (1) higher computational efficiency because of tensor computations and parallelization*
>
> ***EVIDENCE:*** *In Fig. 8, large gains in computation time are seen. Since at the core, the gains are achieved by relying on the Einstein summation representation, it would be interesting to expand on this in section 3. It is neat that the scripts producing the figures are included and the figures can all be reproduced for run-time comparisons. The authors list pacpy (which seems not actively supported anymore) and pactools as comparisons to their toolbox. As pactools also offers parallelization, it is of interest to compare potential speed gains directly to pactools, not only vector-based usage in a loop of tensorpac functions.*

We do agree with the reviewer's comment that a comparison between the vector-based usage loop and the tensor implementation is informative but not optimal. Therefore, as suggested by the reviewer, we ran a direct comparison between Pactools and Tensorpac. To this end, we simulated 4 scenarios :

[8] https://github.com/EtienneCmb/tensorpac/tree/master/paper

1. single-core computations without surrogates
2. multi-cores without surrogates
3. single-core with 20 surrogates
4. multi-cores with 20 surrogates

In each scenario, we computed a comodulogram (20 trials; 4000 time points; 50 phases; 40 amplitudes). Across all four scenarios, Tensorpac performs better than Pactools with sometimes very large differences (e.g PLV). This was the case for all comparisons, except when using the MVL in a single-core context without surrogates.

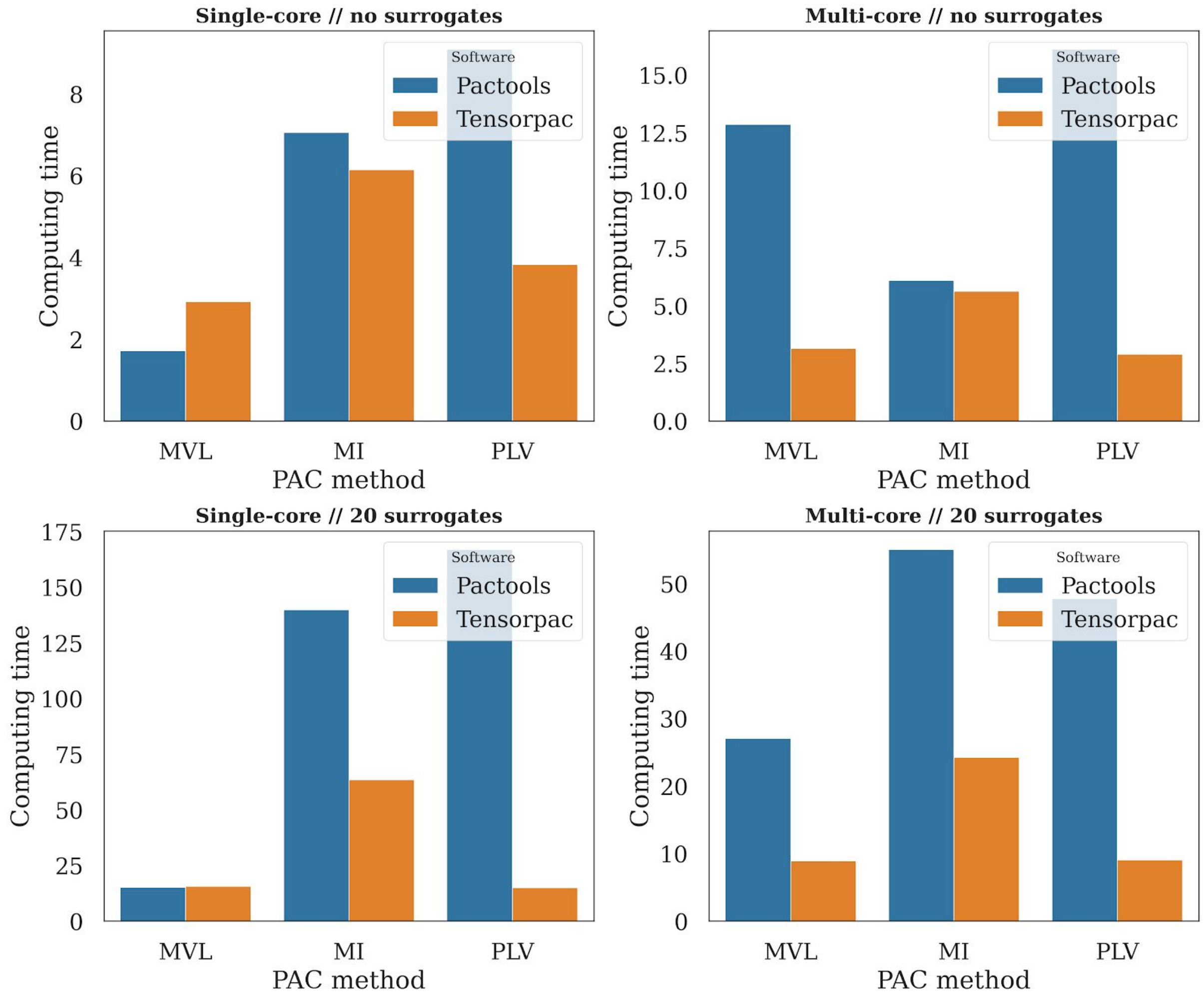

This said, even if these results highlight the computational advantage of Tensorpac compared to Pactools, we believe there are good reasons not to include this result in the manuscript. The first one is that computing the comodulogram also includes the extraction of the phases and amplitudes. Which means that here, we are not directly addressing the computational gains of using the tensor-based implementations of the PAC methods. A second reason, and this is to be completely transparent with the reviewer, using lower requirements (i.e decreasing the number of phases and amplitudes) makes often faster, although the differences were in the order of milliseconds. This was particularly true in the case of single-core computations

without surrogates. Furthermore, we can not guarantee the sustainability of these results on a different machine, with a different version of Python, NumPy, a different number of cores etc. The last reason is that we don't want to initiate a competition with Pactools, because both packages have strengths and weaknesses and are complementary. However, because we agree that this is an important issue, the script for reproducing this figure above is also included in Tensorpac. This allows any user to measure and compare the computational time using the two softwares.

> *(2) the implementation of all most widely used PAC methods in one package*
>
> ***EVIDENCE:*** *According to documentation of pactools, there also numerous methods to calculate the comodulogram are implemented, even more than tensorpac, e.g. driven auto-regressive methods and bispectra, which makes the statement that other packages offer a 'limited choice of PAC computations' not backed up. A more elaborate discussion of the criteria for selecting the chosen methods implemented in 'tensorpac' and highlighting improvements over existing packages would be helpful.*
>
> *The authors stay agnostic to which metric should be used, but possibly there are cases for which each metric has advantages (briefly alluded to in section 1.4) and the brief descriptions of the metrics could be expanded in that respect.*
>
> *From the API documentation, there is also a Gaussian Copula Mutual information PAC/ERP-PAC function, but it is not discussed in the paper, maybe it would be of interest to include in the manuscript.*

Yes, it is true that Pactools offers a larger number of PAC methods computed across time-points. In order to be more accurate, we changed the sentence "*limited choice of PAC computations***" for :**

"While the available tools are extremely valuable, and although some of them provide multiple methods for PAC computation, existing tools do not always include time-resolved PAC measurements and often have limited options for visualization and statistical assessment."

However, Tensorpac also includes two additional methods for estimating event-related PAC, still tensor-based, computed across trials (ERPAC, Voytek et al. 2013, as well as the Gaussian-Copula ERPAC), which to the best of our knowledge, represent a unique feature across existing PAC Python packages.

Regarding the choice of the method, and as the reviewer said, it is hard to make a recommendation since there is, to the best of our knowledge, no gold standard method in the literature. In addition to this point, Tensorpac aims to provide a common platform for researchers to centralize old and recents developments of PAC methods thanks to a modular implementation. That said, it is true that by construction there are methods that should be avoided (in particular those that are amplitude

dependent). Therefore, we added a sentence at the end of the *1.3 Implemented PAC methodologies* **section to make some overall recommendation :**

“While there is still no gold standard for choosing the most appropriate PAC method, some of them are by construction less suitables for measuring genuine coupling. In particular those for which an increase of power would also lead to an increase of coupling (amplitude dependency). Therefore, we recommend choosing methods like the MI, HR or gcPAC which are all not affected by the magnitude of amplitude..**”**

As far as the choice of the method to computing surrogates is concerned, the original manuscript already relays the recommendation of Aru et al. 2015 in the section *1.4 Statistical analysis of PAC* **:**

“*The latter method, with only two blocks, has been described as the most conservative strategy to generate the distribution of PAC that can be observed by chance [47]*”

Indeed, Tensorpac also includes a Gaussian-Copula PAC and ERPAC, which quantify the information shared by the phase and the amplitude, either across time-points or across trials. Since the two reviewers 2 and 3 made the same suggestion, we decided to include it in the method description such as in the figures (Fig3 and Fig5). We also added this new section describing the Gaussian-Copula PAC to the revised manuscript :

“**Gaussian-Copula PAC :** Mutual Information (MI) can be used to quantify pairwise statistical dependence between many different types of variables on a common and meaningful effect size scale (bits) (Cover & Thomas, 1991). However, MI can be difficult to estimate in practice as it requires sampling the full joint distribution of the two considered variables, each of which can themselves be multivariate. Gaussian-Copula Mutual Information (GCMI) is a recently proposed semi-parametric estimation technique (Ince et al. 2017) which has some advantages for estimating MI from neural data. GCMI exploits the fact that mutual information is copula entropy: MI does not depend on the marginal distributions of the variables, but only on the copula function which describes their dependence. GCMI therefore first transforms the inputs to be standard normal. This copula-normalisation step involves calculating the inverse standard normal cumulative density function (CDF) value of the empirical CDF value of each sample, separately for each input dimension, before using a parametric, bias-corrected, Gaussian MI estimator. This provides a lower bound estimate of the MI, without making any assumption on the marginal distributions of the input variables. GCMI is rank-based, robust and scales well with multidimensional variables as the Gaussian model reduces the curse of dimensionality that faces other methods such as those involving binning.

In the case of PAC, the phase $\Phi(t)$ and the amplitude $a(t)$ are still extracted using the Hilbert transform or using wavelets. The rank normalization is first applied on the amplitude $a(t)$. To preserve the cyclic nature of phase, it is represented as points on the unit circle of the complex plane, represented as a 2d variable for GCMI. This 2d variable is built by concatenating the sine and cosine of the phase $\Phi(t)$, $([sin(\Phi(t)), cos(\Phi(t))])$ projecting it into

the unit circle (or alternatively by normalising away the amplitude of the complex value). The copula normalization procedure is then applied to each dimension of this 2d variable. PAC is measured as the GCMI between the copula-normalized 1d high-frequency amplitude and the 2d representation of low-frequency phase :

$$gcPAC = I(\, a(t);\ [sin(\Phi(t)),\ cos(\Phi(t))]\,)$$

Since the gcPAC is rank-based, it is, by construction, invariant to overall amplitude shifts which means that a power increase does not lead to an increase of coupling. Note that measuring PAC using mutual-information has already been proposed using nearest-neighbour estimators (Martínez-Cancino et al., 2019). Similarly to the other measures, Tensorpac provides a tensor-based implementation of the GCMI computed between two continuous variables."

> *(3) advantages for the statistical analysis of PAC measures*
>
> *EVIDENCE: the authors provide methods for computing signal stationarity and surrogate null distributions. An often-desired outcome of PAC analysis is to derive a significance level of obtained comodulogram values, disentangling genuine PAC from spurious PAC. It would be helpful to provide discussion of it in the article (some functions for inferring p-values are present in the toolbox), as well as treatment of multiple comparison correction (cluster-based?) and overfitting & hyperparameter selection, for instance in the context of the proposed triangular search for optimizing frequency intervals.*

We thank the reviewer for this statistical insight. Indeed, Tensorpac includes the inference of p-values using the null-distribution. We added the following paragraph to the section *1.4 Statistical analysis of PAC* **into the revised manuscript :**

"Finally, the null distribution can also be used in order to infer the p-value. Indeed, the p-value is defined as the proportion of surrogates that are exceeded by the true value of coupling. For a comodulogram that contains several phase and amplitude frequencies, Tensorpac also includes a correction for multiple-comparisons. By default, it uses the maximum statistics which provides a threshold that controls family-wise error rate (Holmes et al., 1996; Nichols and Holmes, 2002). The procedure consists in taking the maximum of the surrogates over all of the computed phases and amplitudes and using this distribution of maximums to infer the p-value."

About the correction using cluster-based statistics, we added an illustrative example[9] to the online gallery of examples, for comparing two conditions with a cluster-based correction for multiple comparisons using the MNE-Python package.

9 https://etiennecmb.github.io/tensorpac/auto_examples/stats/plot_compare_cond_cluster.html#sphx-glr-auto-examples-stats-plot-compare-cond-cluster-py

> (4) extended PAC visualization capabilities.

EVIDENCE: *the toolbox provides visualization of comodulograms, frequency-amplitude histograms and polar plots, which are easy to produce and helpful visualizations. As a user, I would find peak/trough-locked (to slow oscillation) time domain & time-frequency plots useful in addition.*

We would like to thank the reviewer for this positive feedback about the included visualizations. Indeed, peak-locking representations offer a simple visualization to see the emergence of PAC at a given phase. To address this request, we added a new class to tensorpac in order to compute and plot the peak-locking (see tensorpac.utils.PeakLockedTF[10] and an illustrative example[11])

Minor

- the '<->' arrow could be substituted by a proper arrow for increased readability

Done.

- there is some inconsistency in referring to KL-divergence. 'KL-distance' 'distance of Kullback-Leibler'

Corrected.

- in the context of PAC, changing the labels/subsections from KLD to Modulation index could be considered for familarity

Agreed. We have now changed everywhere the PAC was called "KLD" to Modulation Index (MI), which uses the Kullback-Leibler distance. The name was also changed in the figures.

- p.6 and following, sometimes there is no space after lots of formulas 'MIformula' 'amplitudes Pdiverges'

Yes, we faced conversion issues between google doc, libreoffice and word. We corrected the missing spaces in the formulas of the revised manuscript.

10 https://etiennecmb.github.io/tensorpac/generated/tensorpac.utils.PeakLockedTF.html#tensorpac.utils.PeakLockedTF

11 https://etiennecmb.github.io/tensorpac/auto_examples/misc/plot_align_tf_pha_peak.html#sphx-glr-auto-examples-misc-plot-align-tf-pha-peak-py

- sometimes an abbreviation is introduced, only to be used once or twice (e.g. PLV, PS, etc) -> use full name throughout for better readability?

This has been corrected in the revised manuscript.

> - Fig.3: I would recommend using the full name of the measure in the subplot titles. Also, here it is noticeable that the scale of the measures is different, maybe the authors could comment on boundedness or ranges of measures in the description of the measures

We updated the Fig3 with the full name of each method and in addition, the reference to the paper. Note that, as Reviewer 1 suggested, the name of the "Phase Synchrony (PS)" method has been changed to "Phase Locking Value" which is more commonly used in the litterature.

> - figure labels and legends are small at times

We checked all of the x-y labels, titles such as colorbar labels to make them larger.

> - p.6 'Height-Ration' -> Height-Ratio

Corrected.

Software and website

> - the toolbox has a clearly indicated license (BSD-3) and I was able to install the toolbox via pip without errors, dependencies are clearly stated.

We are pleased to hear that the installation went well. Note that in the new Tensorpac version, some examples now use MNE-Python and some internal functions can benefit from the installation of Numba. We made this clear from the installation section in the online documentation.

> - statement of need: The high-level description on the documentation page could be expanded to highlight the speed gains by using tensor so they are clearer at first sight. (I think it is a nice selling point)

Yes, this is a great idea. As the figure illustrating the computational benefits of tensor-based implementation over a loop implementation is included in the manuscript, we added a figure to the first page of the documentation[12] that illustrates the idea and the concept of computing PAC with tensors.

[12] https://etiennecmb.github.io/tensorpac/index.html#tensor-based-implementation

> - tutorial link: at the moment there is only a GIF 'under construction'... it would be good to have just a simple example placed there, maybe the one from the nice Fig.1 illustration? Or delete this link and have only the examples page?

We moved the tutorial empty page to the gallery of examples since it can now be updated with recent commits and therefore always be up-to-date. Note that, following a recommendation by another reviewer, the tutorial section[13] now includes a full tutorial for conducting PAC analysis using real data (Combrisson et al. 2017).

- examples: The examples include nice illustrations, but the high-level text could be extended to clarify the purpose of the example, e.g. for "Compute p-values" the text only says: 'For the visualization, we used a comodulogram.', for 'Compare surrogate methods' -> 'Surrogates are used to generate a chance distribution in order to correct the PAC estimation.'. Extension to something like 'This example illustrates how …' would be informative for users, especially novice.

We thank the reviewer for highlighting this lack of explanations in the examples. We updated several examples with more detailed descriptions. Here are the examples that we updated :

- **Example illustrating how to compute the p-values[14]**
- **Example illustrating the comparison between implemented PAC methods[15]**
- **Example illustrating the comparison between methods for assessing surrogates[16]**
- **Example illustrating the comparison between normalization types[17]**

[13] https://etiennecmb.github.io/tensorpac/auto_examples/index.html#tutorials

[14] https://etiennecmb.github.io/tensorpac/auto_examples/stats/plot_pvalues.html#sphx-glr-auto-examples-stats-plot-pvalues-py

[15] https://etiennecmb.github.io/tensorpac/auto_examples/pac/plot_pac_methods.html#sphx-glr-auto-examples-pac-plot-pac-methods-py

[16] https://etiennecmb.github.io/tensorpac/auto_examples/pac/plot_compare_surrogates.html#sphx-glr-auto-examples-pac-plot-compare-surrogates-py

[17] https://etiennecmb.github.io/tensorpac/auto_examples/pac/plot_compare_normalizations.html#sphx-glr-auto-examples-pac-plot-compare-normalizations-py

> - the API documentation is too brief. For instance, the documentation for the 'ndpac' method just reads "Normalized direct Pac." or 'swap_pha_amp' : "Swap phase / amplitude trials." It would be good to include a brief rationale for each function that would clarify what happens to the input.

Indeed, the description of the functions that are used in order to estimate the distribution of PAC that could be obtained by chance was not sufficiently clear. In the revised version, we updated the description of all PAC functions as well as those for computing surrogates. [Here's the link to the API of the updated descriptions](https://etiennecmb.github.io/tensorpac/api.html#surrogates-methods)[18]

> - the main PAC methods have very short function names, possible it could enhance readability giving them slightly more descriptive names? e.g.: hr -> heights_ratio, ps -> phase_synchrony etc

We have now changed the names of the methods to have a more explicit name :

- **mvl() ➝ mean_vector_length()**
- **kld() ➝ modulation_index()**
- **hr() ➝ heights_ratio()**
- **ndpac() ➝ norm_direct_pac()**
- **ps() ➝ phase_locking_value()**

> - some of the function names are in camel case (BinAmplitude) and some have snake_case, unification would be good.

Actually, BinAmplitude, PSD, Pac and ITC are all classes and not functions. We opted for classes because plotting methods could be inserted inside and from the user side it is just more convenient. This is why these names are in camel case.

> - testing: the tests currently included test basic input output relations (smoke tests), but it would be helpful to include functional tests of PAC computation for synthetic signals, for which the ground truth is known.

We thank the reviewer for this very relevant feedback. Indeed, we were not aware of the difference between smoke tests and functional tests. As recommended by the reviewer, we included a functional test for the PAC (see tensorpac.tests.test_pac[19]).

For testing the PAC we added a method called *test_functional_pac()*. As recommended by the reviewer, we generated a ground truth coupling between a 10hz phase and a 100hz amplitude. Then, we compute the comodulogram and the p-values and we detect everywhere PAC measures are significant (p<0.05). As a result, a boolean array is returned (*is_coupling*), where True stands for significant coupling, and then compared with the ground truth boolean array (*gt*). The test passes if 95% of the pixels are correct. In statistical terms, this test is called the "Accuracy" since it

[18] https://etiennecmb.github.io/tensorpac/api.html#surrogates-methods
[19] https://github.com/EtienneCmb/tensorpac/blob/master/tensorpac/tests/test_pac.py

measures the overall detection of both true positives (TP) and true negatives (TN) effects divided by the total number of pixels (accuracy=TP + TN / FP + FN + TP + TN). Please find below the figure output of the test :

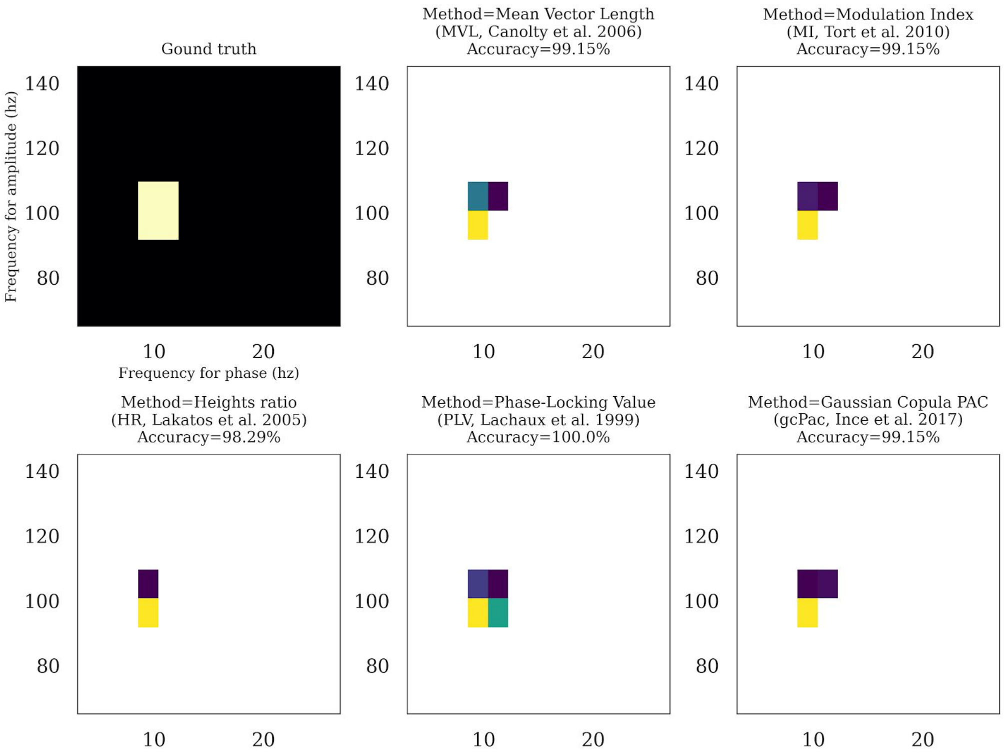

We also included a functional test for the Event Related PAC (ERPAC - ***test_functional_erpac()***), following the same approach as above. However, this time the accuracy was below the 95% accuracy (~70-80%). Therefore, we fixed an accuracy threshold of 80% which we think is really reasonable. One possible reason could be that ERPAC is computed across trials and not time-points and is probably much more sensitive to noise. Probably even more importantly, this method involves an ERPAC estimation at each time-point and the included statistics do not take into account the frequency-temporal structure. For sure, the statistical power provided by a cluster-based approach would probably fix this lower accuracy issue at a non-negligible computational cost.

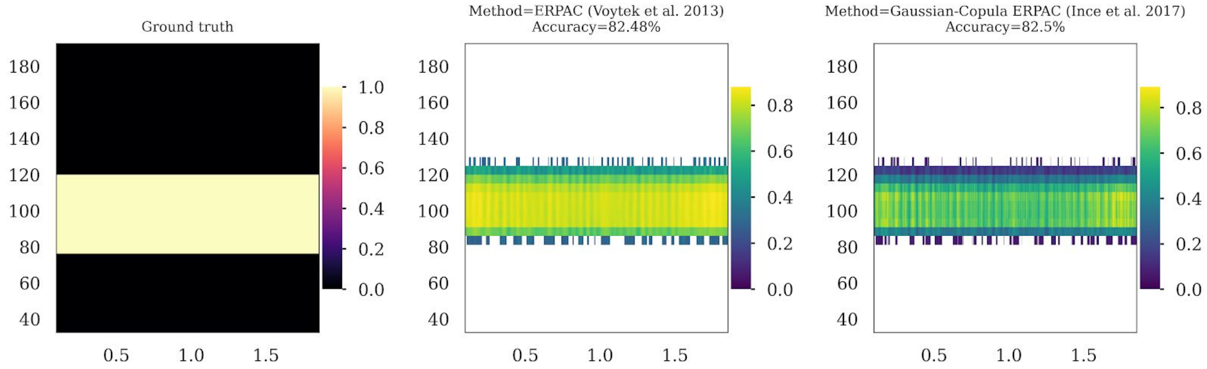

> - paper figures scripts: I needed to adjust the path in other to reproduce the figures from the paper in order for the script not to crash; the path could be changed to a relative one, so this does not happen

We changed the organization of the code for reproducing the figures of the paper (as well as the figures produced for the response to the reviewers) in such a way that it is now using a relative path. Thanks for the suggestion.

> - reference for mvl method is cut short in the API doc

We completely changed the way citations and references are managed in the documentation. We switched to sphinx bibtex to have a proper bib file that contains all of the papers and there is now a "Reference" link (which appears at the bottom of each page in the online documentation).

We sincerely thank this reviewer for all the helpful and detailed comments on the manuscript and the software.
